# Clinical impact of a commercially available multiplex PCR system for rapid detection of pathogens in patients with presumed sepsis

**DOI:** 10.1186/1471-2334-9-126

**Published:** 2009-08-11

**Authors:** Christine Dierkes, Boris Ehrenstein, Sylvia Siebig, Hans-Jörg Linde, Udo Reischl, Bernd Salzberger

**Affiliations:** 1 Department of Internal Medicine I, University Hospital Regensburg, 93042 Regensburg, Germany; 2 Institute of Medical Microbiology and Hygiene, University of Regensburg, 93042 Regensburg, Germany

## Abstract

**Background:**

Timely identification of pathogens is crucial to minimize mortality in patients with severe infections. Detection of bacterial and fungal pathogens in blood by nucleic acid amplification promises to yield results faster than blood cultures (BC). We analyzed the clinical impact of a commercially available multiplex PCR system in patients with suspected sepsis.

**Methods:**

Blood samples from patients with presumed sepsis were cultured with the Bactec 9240™ system (Becton Dickinson, Heidelberg, Germany) and aliquots subjected to analysis with the LightCycler^® ^SeptiFast^® ^(SF) Test (Roche Diagnostics, Mannheim, Germany) at a tertiary care centre. For samples with PCR-detected pathogens, the actual impact on clinical management was determined by chart review. Furthermore a comparison between the time to a positive blood culture result and the SF result, based on a fictive assumption that it was done either on a once or twice daily basis, was made.

**Results:**

Of 101 blood samples from 77 patients, 63 (62%) yielded concordant negative results, 14 (13%) concordant positive and 9 (9%) were BC positive only. In 14 (13%) samples pathogens were detected by SF only, resulting in adjustment of antibiotic therapy in 5 patients (7,7% of patients). In 3 samples a treatment adjustment would have been made earlier resulting in a total of 8 adjustments in all 101 samples (8%).

**Conclusion:**

The addition of multiplex PCR to conventional blood cultures had a relevant impact on clinical management for a subset of patients with presumed sepsis.

## Background

Early adequate antibiotic treatment improves the outcome of patients with sepsis [1-5]. Even if broad spectrum antibiotics are used empirically, adjustments of antimicrobial therapy may be necessary. Generally adjustments are based on the results of positive blood or other cultures that are available after 8 to 48 hours [6].

Additionally, the likelihood of a positive result in conventional culture methods can be reduced by concomitant or prior antibiotic treatment. Amplification of bacterial and fungal nuclear acids directly from blood specimen is a newly established detection method for pathogens. Tests based on this method may improve clinical care by shortening the time to a positive result and by being more independent of antibiotic pre-treatment. The impact of these methods on therapeutic decisions and outcome has not yet been studied.

We compared the results of a standard BC system with those of a commercially available polymerase chain reaction (PCR) based system in routine clinical management.

Primary goal of the study was to determine if results from the additional PCR based system led to different therapeutic decisions than the results from BC alone.

Secondary goal was to assess the concordance of both methods and time saving effects with the use of a PCR-based test.

## Methods

A commercially available molecular based test system (LightCycler^® ^SeptiFast^® ^(SF) Test; Roche Diagnostics, Mannheim, Germany) was offered as an add-on diagnostic tool free of charge supplemental to standard BCs taken in patients with presumed sepsis at the Regensburg university medical centre during an eight months period (July 2006 – March 2007). The test was made available to all departments treating patients with sepsis by the microbiology department. The decision to use the additional test was solely made by the treating clinician.

The SeptiFast^® ^Test is able to detect 25 different pathogens directly from blood by real-time multiplex PCR (Table 1) [7]. The limits of detection were 100 CFU/ml for coagulase-negative staphylococci, *C. glabrata *and Streptococcus spp. and 30 CFU/ml for all other pathogens listed in the table [8].

**Table 1:** SeptiFast^® ^Portfolio: Pathogens detected by SeptiFast^®^.

Gram-negative bacteria	Gram-positive bacteria	Fungal pathogens
*Escherichia coli*	*Staphylococcus aureus*	*Candida albicans*
*Klebsiella pneumoniae*	*Coagulase-negative*	*Candida tropicalis*
*Klebsiella oxytoca*	*Staphylococci*	*Candida parapsilosis*
*Serratia marcescens*	*Streptococcus pneumoniae*	*Candida krusei*
*Enterobacter cloacae*	*Streptococcus spp.*	*Candida glabrata*
*Enterobacter aerogenes*	*Enterococcus faecium*	*Aspergillus fumigatus*
*Proteus mirabilis*	*Enterococcus faecalis*	
*Pseudomonas aeruginosa*		
*Acinetobacter baumannii*		
*Stenotrophomonas*		
*maltophilia*		

Both the sample for the BC and the sample for the SF were taken through the same blood drawing device. Both aerobic and anaerobic BC bottles (Bactec 9240 BC system, Becton Dickinson, Heidelberg, Germany) were inoculated directly with 10 ml blood each and delivered to the microbiology department together with the aliquot for analysis with the SF. Blood cultures were incubated for 7 days. The SF-test was provided free of charge by the manufacturer.

A retrospective analysis of the test results and clinical data was performed. Clinical data were extracted by chart review (underlying illness, antibiotic pre-treatment, epidemiological data such as age, sex, length of stay, hospital mortality, immunosuppression, SIRS criteria (Temperature ≤ 36°C or ≥ 38°C, heart rate ≥ 90 bpm, respiratory rate ≥ 20 breaths/min or paCO_2 _< 32 mmHg, white blood cell count ≥ 12,000 or ≤ 4,000 cells/mm^3^)) and by the data provided for the test application. Immunosuppression was defined as being post organ-transplant, receiving chemotherapy for malignant disease or receiving high dose prednisolone (>20 mg/day). Those with chronic disease predisposing for infections such as chronic liver disease or alcoholism were not counted as such. Antibiotic pre-treatment was defined as having received any antibiotic therapy within 24 hours before specimen collection. Multiple samples per patient were allowed, if the times of blood collection were at least 48 hours apart.

To determine the impact of the results on the patients' therapy, data were independently reviewed by two infectious disease specialists. They analyzed the current antibiotic therapy and possible adaptations necessary for optimal treatment according to current standard of care. In case of both SF and BC yielding positive results, the possible time saving effect of the SF-test was determined.

Treatment adjustments were classified into two different categories: 1) Treatment adjustment not necessary (either due to coverage by the empirical therapy or to lacking clinical relevance); 2) Treatment adjustment necessary due to lacking coverage of empirical therapy.

In order to examine possible time saving effects of the molecular based analysis, time to results of BCs was compared to the time of SF results. At our institution BC results are reported to the treating physician by telephone call from 8 am to 7 pm Monday-Friday and 9 am – 4 pm on weekends and holidays. A complete report including antibiotic resistance testing results is faxed and sent by mail. Due to the experimental status of the SF-test, the results were not available on a daily basis. Therefore a virtual once and twice daily analysis Monday-Friday was assumed in order to achieve comparable time frames. For the once daily simulation results were assumed to be available by 2 pm if received by 8 am in concordance with the turnaround times reported for the test. In the twice daily setting results were assumed to be available by 2 pm if SF samples were received by 8 am, if received by 1 pm results were assumed to be available at 7 pm. We then compared the time from drawing the blood samples to the time a first positive BC result was reported and the virtual SF result time.

Data collection and statistical analysis were performed using Microsoft Office Access^® ^(Version 2007, Microsoft Corporation, Redmond, USA) and SPSS^® ^(SPSS Inc, Version 15, Germany).

Due to the retrospective and non-interventional design of the analysis approval of the local ethics committee was not required according to the guidelines of our hospital.

## Results

### Patient population

In the study period 101 samples of 77 patients were examined. The median age of the patients was 55 years (Table 2). In-Hospital mortality was 33% with a median length of stay of 35 days. Eighty-three patients (82%) were treated in the ICU while the blood samples were taken. Two or more SIRS criteria were present in 79 patients at the time of specimen collection (78%), one positive SIRS criteria in 13 patients, no SIRS criteria in 8 patients (missing data in one patient). Thirty-five of the patients (45%) were immunocompromised. In 40 patients from surgical and 37 from medical wards a broad spectrum of diseases and comorbidities was present. Concomitant antibiotic therapy at the time of specimen collection had been administered in 61 patients with 83 samples (81%) studied.

**Table 2:** Patient characteristics: Patient characteristics stratified by SeptiFast^® ^(SF) results.

	SF positive			SF negative	
	
	SF and BC detected same pathogen	Only SF detected pathogen	SF and BC detected different pathogens	BC detected pathogen	BC and SF neg.
**Number of samples**	14	12	3	9	63
					
**Number of patients**	12	8	2	7	48
					
**Patients in ICU at time of specimen collection**	11	11	2	9	50
**Number of samples drawn with concomitant antimicrobial therapy**	13	12	2	7	50
**Median Age** **(± SD) [years]**	59 (± 18)			52 (± 17)	
					
**Gender**	8 female/14 male			20 female/35 male	
					
**Median Duration of hospitalisation (± SD) [days]**	40 (± 39)			34 (± 43)	
					
**Hospital mortality**	36.4%			30.9%	
					
**Median Number of SIRS criteria (± SD)**	2.9 (± 1.1)			2.2 (± 1.1)	

BC = blood culture.

### Isolated Pathogens

In 39 samples (38%) pathogens were identified from either BC or SF (Table 3). Both methods yielded predominantly gram-positive bacteria. More pathogens were found in the SF group of patients, mainly due to a higher rate of fungal pathogens and a higher rate of *Enterococcus faecium*, while the number of coagulase-negative Staphylococci (CNS) was lower with SF. All differences were not statistically significant (p > 0.05).

**Table 3:** Isolated Pathogens: Pathogens identified in BC or SF; 7 samples yielded polymicrobial results.

Species	SeptiFast^®^	Blood culture
Coagulase-negative staphylococci	3	6
*Staphylococcus aureus*	5	3
*Enterococcus faecalis*	2	3
*Enterococcus faecium*	7	3
*Klebsiella pneumonia*	2	1
*Serratia marcescens*	2	2
*Escherichia coli*	3	2
*Enterobacter cloacae/aerogenes*	2	1
*Proteus mirabilis*	1	1
Streptococcus species	1	
*Candida albicans*	2	3
*Candida glabrata*	1	
*Aspergillus fumigatus*	1	
*Candida tropicalis*	1	1
*Candida krusei*	1	
*Streptococcus pneumoniae*		1
*Listeria monozytogenes*		1

**Total**	**34**	**28**

### Concordance of BC and SF

Fourteen of 101 samples (13%) were concordant positive in SF and BC systems. Sixty-three (62%) were negative in both methods. Thus a concordant result was seen in 77% of all samples. In 13 samples with a negative BC, pathogens were identified by SF (Table 4). In additional microbiological samples from the same patient, the identical pathogen was isolated in 9 of those cases (bronchial lavage (3), wound swab (2), tracheal aspirate (1), urine (1), ascites (1), gall fluid aspirate (1)); Table 5.

**Table 4:** Overall results: Results of blood culture (BC) and SeptiFast^® ^(SF)

	BC positive	BC negative	Total SF
**SF positive**	14	13	**27**
**SF negative**	9*	63	**73**
**Total BC**	**23**	**77**	**100^#^**

* Two samples yielded two different pathogens by BC but just one by SF

# One sample yielding different species comparing SF with BC not included in table.

**Table 5:** Additional microbiological samples: Pathogens identified by culture from patients with positive SF and negative BC result.

Species	Organ Site
*Enterococcus faecium*	Wound swab
*Enterococcus faecium*	Gall fluid aspirate
*Enterococcus faecium*	Ascites
*Enterococcus faecium*	Tracheal aspirate
*Escherichia coli*	Urine culture
Streptococcus species	Wound swab
*Candida albicans*	Bronchial lavage, Skin swab groin
*Candida krusei*	Bronchial lavage
*Aspergillus fumigates*	Bronchial lavage

Fungal pathogens were detected more frequently with the SF-test when present in other clinical isolates (3 *Candida *species). *Enterococcus faecium *infection was more often diagnosed in the SF (n = 7) test compared to BC (n = 3), although neither difference was statistically significant.

In 9 samples with positive BC the SF-test yielded negative results (Table 6). *Listeria monozytogenes *isolated from a BC was not detected by the SF-test due to the limited portfolio of the SF-test. Another failure was attributed to technical problems during the analysis. Three times CNS were isolated by BC while SF did not give a positive result. Four further samples with positive BC results had no positive result in the SF analysis. Three of those were polymicrobial bloodstream infections with two samples with *Enterococcus faecalis *that failed detection and one *Streptococcus pneumoniae *bacteraemia. Retrospective analyses of the original data of the LightCycler^® ^system revealed the detection of *Streptococcus pneumoniae *that had been missed due to a faulty software (V1.1.7., detected in review with revised software, V1.2.1).

**Table 6:** Negative SF and positive blood culture: Isolated Pathogens per Patient with negative SF and positive blood culture result

Pathogen	Note
Candida albicans	BC positive after 5 days
Streptococcus pneumonia	Pathogen identified when looking retrospectively at Lightcycler^® ^data
*Enterococcus faecalis, Coagulase-negative staphylococci*	Unknown
*Coagulase*-negative staphylococci	Suspected contamination
*Coagulase*-negative staphylococci	Suspected contamination
Candida albicans	Not enough blood for SF (750 μl instead of 3 ml)
*Enterococcus faecalis*, Coagulase-negative staphylococci	Unknown
Listeria monocytogenes	Not included in SF portfolio, Ampicillin added
E. cloacae	E. cloacae found, E. faecium missed

The median time to obtain the result of the SF analysis was determined to be 18 hours, with a minimum of 6.75 hours and a maximum of 74 h (samples collected at the beginning of the weekend) in the twice daily situation, with a median time of 26,25 hours, a minimum of 6,75 hours and a maximum of 79 hours assuming a once daily analysis.

### Treatment adjustments

In samples with both BC and SF positive a change in therapy would not have been necessary in 11 of the 14 cases (Table 7). For one of those samples the BC results had been available before completion of the actual SF analysis and a change in therapy had already been executed.

**Table 7:** Positive blood culture and SeptiFast^®^: Isolated pathogens per patient with positive blood culture and positive SF result

Identified pathogen	Therapeutical relevance
*Escherichia coli*	No change: patient deceased before results became available, empirical therapy sufficient
Coagulase negative staphylococci	Modification necessary: Vancomycin added
*Serratia marcescens*	No change, empirical therapy sufficient
*Staphylococcus aureus*	No change, BC results leading to change were available before SF
*Serratia marcescens*	No change, empirical therapy started after sample collection sufficient
*Enterococcus faecium*	Modification necessary: Vancomycin added
*Klebsiella pneumoniae*	No change, empirical therapy sufficient
*Candida tropicalis*	No change, empirical therapy started after sample collection sufficient
*Staphylococcus aureus*	No change, empirical therapy sufficient
*Proteus mirabilis*	No change, empirical therapy sufficient
*Enterococcus faecalis*	No change, empirical therapy sufficient
*Candida albicans*	Modification necessary: Fluconazole added
*Staphylococcus aureus*	No change, empirical therapy sufficient
*Escherichia coli*	No change: patient deceased before results became available, empirical therapy sufficient

Three samples of 3 different patients could be identified where a treatment adjustment was deemed necessary. In one case an antifungal drug was not included in the empiric regimen; in two other cases vancomycin was necessary additionally. The median time from specimen collection to the SF result was 27 hours in the once daily and 18 hours in the twice daily setting in all BC and SF positive samples. The SF median time in the 3 samples that resulted in treatment adjustments was 21.5 hours for both scenarios (once and twice daily: 7; 21.5; 22 hours) compared to 29 hours median time for BC (29; 8; 43 hours) for these individual cases.

Compared to the BC results of those samples this yields a time saving of a median of 21 hours.

In samples with BC negative and SF positive results a treatment adjustment was not deemed necessary in 8 of 13 samples (Table 7, Table 8). In three cases the additional use of antifungal treatment was recommended. Two other patients had not been treated for *Enterococcus faecium *and vancomycin was recommended as an additional drug.

**Table 8:** Positive SeptiFast^® ^and negative blood culture: Isolated Pathogens per Patient with positive SF and negative blood culture result

Pathogen	Therapeutical relevance
Coagulase negative Staphylococci	No change, empirical therapy sufficient and probable contamination
*Enterococcus faecium*	No change, empirical therapy sufficient
*Enterococcus faecium*	Modification necessary: Vancomycin added
*Enterococcus faecium*	No change, empirical therapy sufficient
*Enterococcus faecium, Klebsiella pneumoniae, Enterobacter cloacae/aerogenes*	No change, empirical therapy sufficient
*Escherichia coli*	No change: patient deceased before results became available, empirical therapy sufficient
*Candida krusei*	No change, empirical therapy sufficient
*Streptococcus *species	No change, empirical therapy sufficient
*Enterococcus faecium*	Modification necessary: Vancomycin added
*Staphylococcus aureus*	No change, empirical therapy sufficient
*Enterococcus faecium, Candida glabrata*	Modification necessary: Voriconazole added
*Aspergillus fumigatus*	Modification necessary: Voriconazole added. Growth of colonies from broncho-alveolar-lavage Aspergillus-Ag elevated.
*Candida albicans*	Modification necessary: Caspofungin added

Taken together, based on our proposed virtual time frame in which SF results would have become available, in 8 of 27 samples an adjustment of therapy would be triggered by the SF results. Three of those would have been made earlier while 5 would have been missed by conventional BC. Thus the use of the SF-test had clinical consequences in 8% (8 of 101) of all analysed samples.

As treatment recommendations were made post-hoc based on the clinical data available at the time of the diagnostic results, we also looked at the actual clinical courses. In all but one sample the treating clinicians had decided in accordance with our post-hoc recommendations.

## Discussion

In 39/101 samples from patients with suspected sepsis pathogens were identified with either the use of BCs or a PCR-based test for the identification of bacterial and fungal pathogens in whole blood (SF-test).

Concordance for positive and negative results between BC and SF (77%) was comparable to other studies [9,10].

With the BC results only, we identified positive results in 21% of all samples in comparison to 27.4% with the SF-test. With SF more fungal pathogens and more *Enterococcus faecium *were identified compared to BC. In contrast, fewer CNS specimens were identified by SF compared to conventional blood cultures.

One important question is if these pathogens additionally detected by either SF or BC are clinically relevant or if there are merely "innocent bystanders", i.e. the important discrimination between contamination, colonisation or true infection. In our patient cohort most pathogens detected only by SF were also present in other specimens of the same patient suggesting a clinical relevance. These results are in concordance with other studies where 12 of 14 respectively 31 of 42 pathogens additionally detected by PCR were present in other samples of the same patient [9,10].

CNS were rarely detected by the SF-test in our collective, as the SF-test tries to omit positive results due to contamination. This is in accordance with one other study, presenting 102 patients without signs of infection with 19.6% positive BC and no CNS by SF [7]. In our study in all patients with CNS identified by BC the antibiotic treatment was not adjusted, alluding to the considered insignificance by the treating clinicians. Whether at least one of them was clinically relevant could not be judged retrospectively.

Our study showed that the use of SF in addition to BC led to the identification of more pathogens. If this was influenced by concomitant antibiotic therapy could not be addressed in our study, as 82% of the patients were already on treatment at specimen collection.

Our assumed twice daily analysis for the SF-test would have led to a median time to final results of 18 hours. Compared to usual turnaround times for BC results, this yields a clear time advantage for the SF-test. Immediate processing of specimens could further shorten the time to final results to 6.5 h [10]. If this approach is feasible in a routine hospital setting has yet to be examined.

Additional pathogens were identified in 5 cases and in 3 samples the rapid SF-test would have led to earlier adjustment of antibiotic therapy. In total the use of the test would have resulted in therapeutic consequences in 8 (8%) patients.

On the other side, 9 pathogens were not detected by SF. One of them is not included in the PCR portfolio, thus no conclusions can be drawn regarding sensitivity. Alarming is the failure of detection of S. pneumoniae, even if this could be attributed to a software problem that was solved in newer software versions. With regard to the missed polymicrobial infections it is unclear why these were not detected by SF. A possible explanation might be the competitive growth in vivo that does not allow both pathogens to reach a level resulting in a positive SF test.

All detection failures and the necessity of antibiotic resistance testing underline the importance of using the SF test additionally to standard BC.

If therapeutic adjustments initiated by the SF-test have an impact on the survival of patients with sepsis could not be determined in this study. Studies indicate that patients do not have a benefit from changes in antibiotic therapy if the initial empiric therapy did not cover the causative pathogen [4]. As those studies are based on the results obtained with current culture systems an average delay of 1–2 days has to be assumed [6]. The effect of shortening this time interval with the use of a PCR test has yet to be examined [11].

Invasive fungal infections have an especially high mortality [4,12,13]. Studies have demonstrated that adverse patient outcomes are related to late or inadequate antibiotic treatment. As the SF-test was superior in detecting fungal pathogens and delivers results more rapidly, the use of SF may improve patient outcomes.

The cost benefit of the SF may differ in different clinical situations or patient populations. Special equipment and specially trained personnel are necessary to perform the test. The cost of individual tests will be influenced by the intended turnaround time as well as by the availability outside normal working hours. Cost and cost-effectiveness analyses will have to be done for different scenarios.

Our study had several limitations. Only 80% of the patients presented with two or more SIRS criteria at the time of the specimen collections. But as the rate of immunosuppressed patients was high, a high index of suspicion was present in the remaining 20%. Additionally, it was a non-randomized study, where the decision to use the additional test was solely taken by the treating clinician. Thus a selection bias has to be assumed that may influence the rate and types of pathogens found in our samples. Further, it is a single centre study with a small study size, thus possibly limiting the generalisability of the results. Nevertheless the design of our study may mirror clinical reality more exact than randomized controlled trials. The patients' characteristics in our study are well comparable to those of patients from sepsis studies or cohorts, including one at our institution [14].

## Conclusion

Our study shows that the use of a rapid molecular diagnostic test for the identification of bacterial and fungal pathogens may lead to a higher rate of early adequate antibiotic therapy in approximately 8% of patients with suspected sepsis. Due to the small sample size and the design of our study cost effectiveness analyses were not feasible. They are warranted in further studies.

## Competing interests

The authors declare that they have no competing interests.

## Authors' contributions

CD participated in the design and coordination of the study, the clinical data acquisition and drafted the manuscript.

BE participated in the design of the study and helped draft the manuscript.

SS participated in the clinical data acquisition and helped draft the manuscript.

HJL participated in the design of the study.

UR participated in the design of the study and carried out the PCR.

SB participated in the coordination of the study, performed the statistical analysis and helped draft the manuscript.

All authors read and approved the final manuscript.

## Pre-publication history

The pre-publication history for this paper can be accessed here:

http://www.biomedcentral.com/1471-2334/9/126/prepub
